# MINIMA Short Stem Versus Standard Profemur (TL) Stem in Primary Total Hip Replacement: A Comparative Study

**DOI:** 10.7759/cureus.23771

**Published:** 2022-04-02

**Authors:** Stylianos Tottas, Athanasios Ververidis, Ioannis Kougioumtzis, Konstantinos Tilkeridis, Christina Tsigalou, Makrina Karaglani, Georgios Drosos

**Affiliations:** 1 Orthopeadics, University General Hospital of Alexandroupolis/Democritus University of Thrace, Alexandroupolis, GRC; 2 Medical-Molecular Microbiology, University General Hospital of Alexandroupolis/Democritus University of Thrace, Alexandroupolis, GRC; 3 Laboratory of Pharmacology, Medical School/Democritus University of Thrace, Alexandroupolis, GRC; 4 Orthopeadics, University General Hosital of Alexandroupolis/Democritus University of Thrace, Alexandroupolis, GRC

**Keywords:** serum markers, minima® stem, femoral components, hip prostheses, short-stem, minimal invasive surgery, total hip arthroplasty, total hip replacement

## Abstract

Background: The objective of our study was to compare a novel squared section, tapered design - with four conicity - short stem, the MINIMA® short stem with the cementless Profemur® TL standard femoral stem in primary total hip arthroplasty (THA) in terms of functional outcomes, radiologic evaluation and other peri-operative and post-operative data.

Material and methods: This is a comparative study including 46 patients undergoing primary THA. In 23 patients, the MINIMA® short stem was used. These patients were matched with another 23 patients in whom a cementless Profemur® TL standard femoral stem was used. The levels of the pain were evaluated according to the Visual Analog Scale/Numerical Rating Scale (VAS/NRS). The functional and clinical evaluation of the patients was performed with Harris Hip Score (HHS), Charnley’s Hip score, EuroQol (EQ-5D)-(EQ-100), Patient Health Questionnaire (PHQ-9), and neuropathic pain questionnaire (DN-4). The rest of the comparison data included demographic data, the American Society of Anesthesiologists score (ASA), Charlson Index score, the pre-operative diagnosis, radiographic evaluation, the days of hospitalization, the operating time, incision length, blood loss, and blood transfusion requirements and complication rates.

Results: The two cohorts had comparable results regarding all patients’ peri-operative data. The radiographic assessment revealed considerable higher levels of femoral offset and femoral subsidence for the MINIMA group, but within acceptable limits for both cohorts. The majority of the functional and other scores did not give strong prominence to one specific femoral stem.

Conclusion: Our comparative study underlined the efficacy of the MINIMA® short stem, due to the fact that it revealed comparable and, in some cases, relatively better short-term outcomes compared with the TL standard femoral stem. Yet, more well-designed long-term research is required in order to further establish its effectiveness.

## Introduction

Total hip replacement (THR) has been described as one of the most successful operations of the 20th century [1], offering relief from pain, improved postoperative functional outcomes, and enhancement of patients’ quality of life [2,3]. Recent evidence reports improved postoperative outcomes regarding clinical results and implants’ survivorship at 10 years follow-up [4-6]. Regardless of the exceptional long-term results of the cemented THR [7,8], the application of cementless implants in THR is being increased constantly with excellent long-term outcomes as well [9-12].

Despite excellent results, some surgeons raise their concern about cementless conventional femoral stems in THR because they may be associated with peri-prosthetic fractures [13,14], thigh pain, and proximal stress shielding. Another contentious issue and probably the most important concern is the loss of bone stock in a possible future revision surgery [15-18].

Over the last two decades, many surgeons have shifted their clinical interest and research on less invasive implants (the so-called short-stems) [18-21]. The implementation of short stems was initiated more than 30 years ago [22,23]. There is no clear-cut answer about the length size of short stems, with many authors indicating their length under 120mm [24].

There are several points worth considering in favor of short stems, including less stress shielding, proximal bone stock preservation, restriction of proximal-distal mismatch and reduced thigh pain [18-20,24]. Recent literature underlines the effectiveness of some of the short stems in terms of survival rate, clinical outcomes and radiologic assessment, which are approximately equal to the conventional stems. However, the majority of the research depicts short and mid-term results [25-28].

This study aims to compare a novel squared section, tapered design - with four conicity - short stem (MINIMA® short stem) and a Profemur® TL classic femoral stem in primary THR patients. The two cohorts were compared in terms of peri-operative outcomes including, clinical, functional, and radiological results. Moreover, we recorded serum and inflammation markers in order to define 1) whether there are differences in bone impairment of the femur between the two stems and 2) whether this fact has any potential interaction with functional and other postoperative outcomes.

## Materials and methods

This is a comparative retrospective study of prospectively collected data concerning 46 patients who underwent THR by the same surgeon (GD) between March 2017 and June 2019. The MINIMA® short stem was implemented in 23 patients and the Profemur® TL classic femoral stem was used in 23 patients as well. All the procedures were performed through the lateral (modified Hardinge) approach.

Our study was approved by the Ethics Committee of the University General Hospital of Alexandroupolis (approval number ΕΣ8/Θ38/22-08-2018). Written informed consent was obtained from all patients.

To compare the two cohorts, patients were evaluated according to various data which were collected prospectively. These data included 1) patients’ demographic in terms of age, gender, body mass index (BMI), 2) possible comorbidities, Charlson comorbidity index [29], the American Society of Anesthesiologists (ASA) score [30], diagnosis (osteoarthritis, dysplasia, osteonecrosis, etc.), 3) peri-operative data regarding the operating time, incision length, the levels of the perceived pain, inflammatory and serum markers, 4) post-operative data including functional outcomes, radiographic assessment, length of hospital stay (LOS), blood loss [31,32], blood transfusion requirements and post-operative complications classified as major or minor, local or systemic [33].

Peri-operative patient’s management

The patients from both cohorts followed the same fast-track program. Initially, all the patients were trained with specific exercises some days before surgery. Thereafter, they were requested to perform these exercises daily in order to become familiar with them. By performing this exercise program post-operatively under the supervision of a physiotherapist as well, the recuperation process was accelerated. Second, a blood management program (BMP) was adopted in our department. Hemoglobin (Hb) was assessed approximately one month pre-operatively and patients with severe anemia were excluded from this study. During surgery, tranexamic acid 2 gr was diluted in 100 mL normal saline and it was administered locally. The transfusion point was defined at 9 g/dL regarding (Hb), taking into account the hemodynamic situation and the vital signs of the patients as well. Third, the pain management program (PMP) was based on various analgesia management including gabapentinoids pre- and post-operatively, paracetamol 1 gr every six hours post-operatively, and non-steroidal anti-inﬂammatory drugs (NSAIDs) (parecoxib 40 mg) in case of elevated pain levels. Intra-operatively, local inﬁltration analgesia (LIA) (ropivacaine 1% in a maximum dose of 3 mg/kg) was also performed. Opioids were used as rescue analgesia. No drains were used. A second-generation cephalosporin (cefoxitin sodium) for 24 h and anticoagulation preventive therapy with low-weight molecular heparin was administered in all patients. Finally, the patients were free to go home just only they fulfilled the same discharged criteria.

Implants

**Figure 1:**
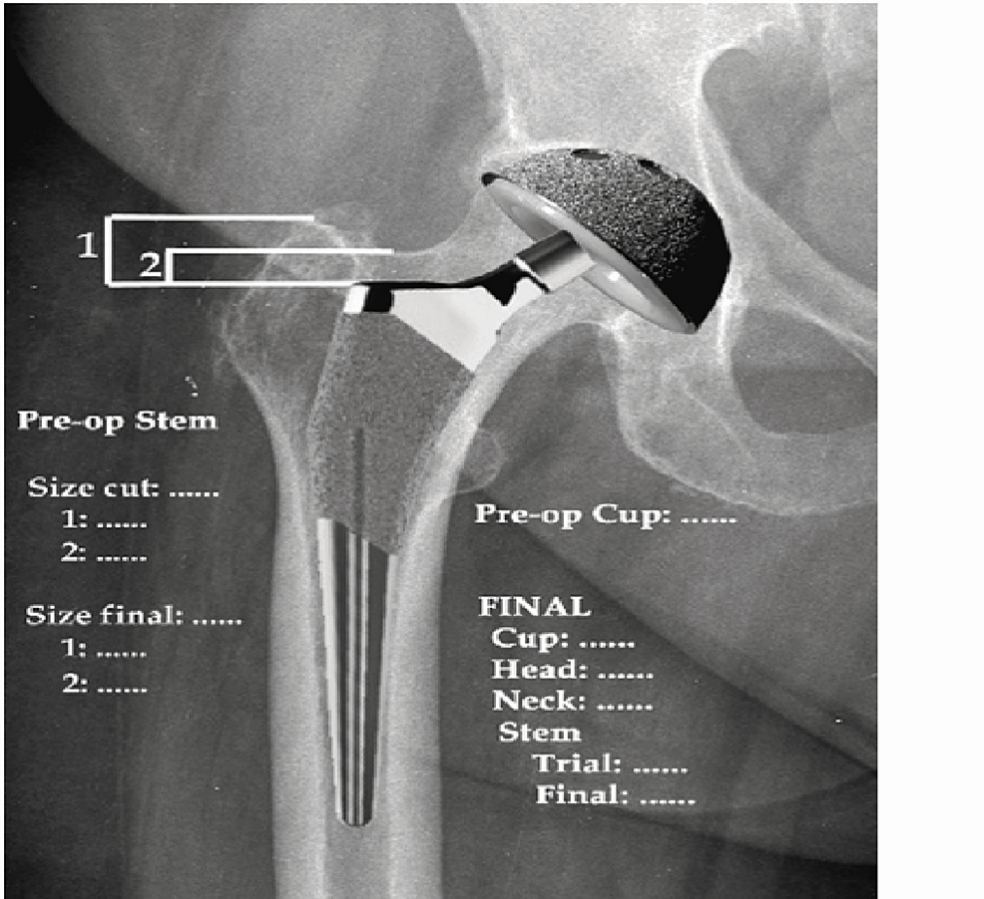
The Profemur® TL standard stem Picture used for the preoperative planning (From the data of professor G. Drosos)

Concerning the conventional stem, cementless Profemur® TL classic femoral stem (Microport, TN) was implemented in 23 patients (Figure 1).

**Figure 2:**
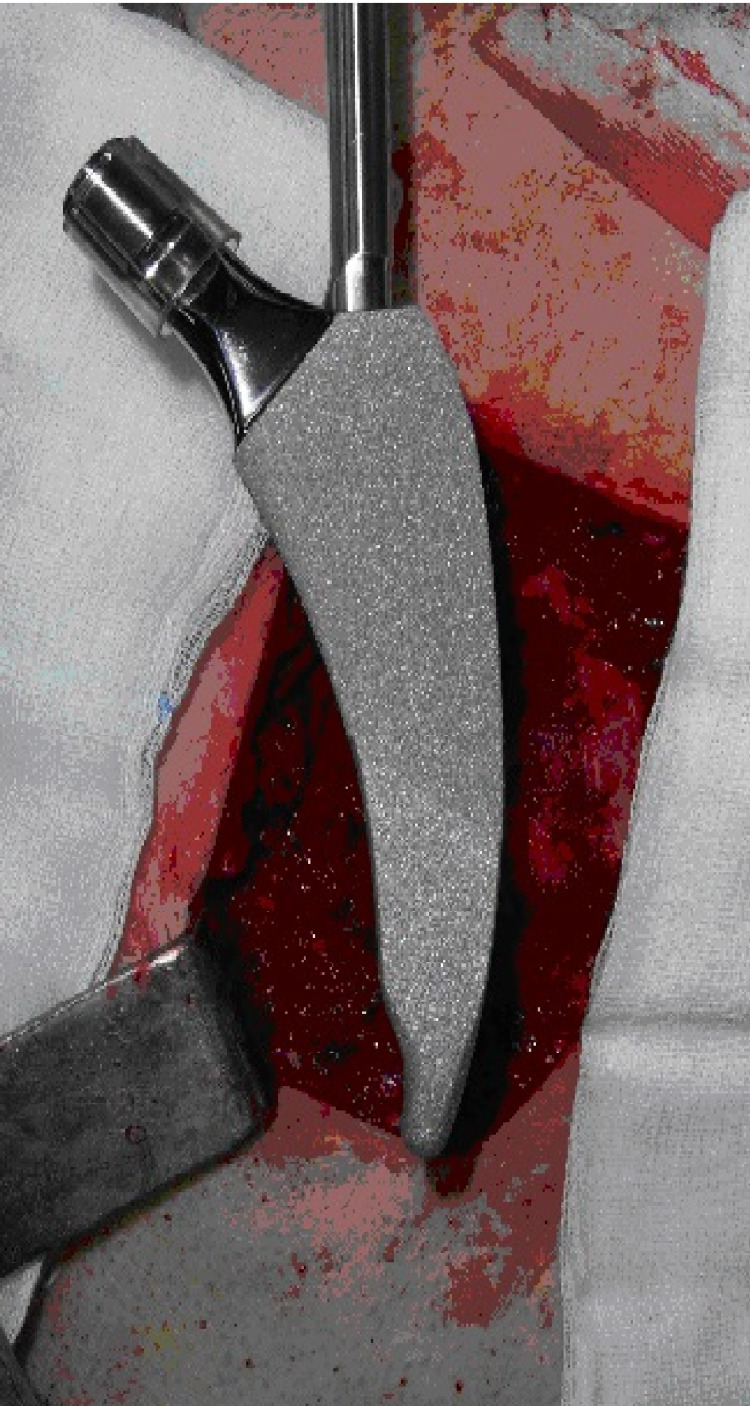
The MINIMA® short femoral stem Intraoperative picture (from the data of Professor G. Drosos)

Furthermore, cementless acetabular components (OHST Medizinetechnik AG, Germany) were used in these patients as well. On the other hand, the MINIMA® short stem (Lima Corporate, Udine, Italy) was implemented in another 23 patients (Figure 2).

It is a novel squared section, tapered design -with four conicity- short stem, available in 12 sizes, with a length range between 82mm and 118mm. This particular stem falls into type 3 category and it is considered a trochanteric sparing stem [34]. Additionally, two cementless acetabulum components were implemented in these patients, 1) Porous Titanium coating - Titanium plasma spray (SPH-Contact® cup, Lima Corp. Udine, Italy) and 2) a Porous Titanium with HA coating (Delta-PF® cup, Lima Corp. Udine, Italy).

As we stated above, this is a comparative study between two different femoral stems in THA without a random selection process.

Radiological evaluation

The digital radiographs were studied and measured using the “IMPAX 6, March 5, 3009” software. The implanted acetabular cup was used for calibration. Patients underwent several regular anteroposterior x-rays of both hips and lateral radiographs of the hip that THA was performed during their follow-up examination. We obtained and studied the radiographs, which were carried out immediately post-operative and at short (one-year) follow-up for each patient. We had the objective to both record the orientation of the components and to assess the radiographic alterations around the components one year post-operatively as well, based on various criteria.

Initially, the acetabular cup anteversion was calculated drawing an ellipse shape which was matched to the border of the cup. The minor and major axes’ ratio defined the range of the cup anteversion [35]. Cup inclination was defined through the angle between the acetabular axis and the longitudinal axis of the body [36]. Leg length discrepancy was estimated using the discrepancy of the distance between the teardrop and the center of lesser trochanter regarding both hip joints [37]. The stem alignment was defined according to the angle between the axes of femoral stem and femoral shaft at the antero-posterior view [38,39]. Femoral offset as the horizontal distance between the center of rotation and the femoral anatomical axis [40].

Subsequently, concerning the acetabular component stability one year post-operatively, we investigated the potential presence of radiolucent lines at the bone-prosthesis interface [41] and the cup stability criteria as stated by Manley et al. [42].

Heterotopic ossification occurrence was registered based on Brooker’s rating system [43]. In addition, femoral stem subsidence was determined as the discrepancy of the intervals between the lesser trochanter apex and the distal tip of the stem instant post-operatively comparing to one year follow-up x-rays [44]. Furthermore, proximal femoral stress shielding and bone resorption were probed, as Engh et al. settled on [45]. Moreover, the femoral stem fixation was fallen into the category of stable bone ingrowth, stable fibrous fixation or unstable fibrous fixation [46]. Another essential radiological feature of our study was the investigation of the modified seven zones of Gruen in anteroposterior and seven zones in lateral radiographs views regarding any eventual radiolucent lines or reactive lines or cortical hypertrophy [47].

Functional and other scores

Functional outcomes were recorded pre-operatively, at six and 12 months post-operatively based on Harris Hip Score (HHS) [48] and Charnley’s Hip score. Simultaneously, general health was evaluated with EuroQol (EQ-5D) - (EQ-100) [49], patients’ depression situation with Patient Health Questionnaire (PHQ-9) and the features of the perceived pain with neuropathic pain questionnaire (DN-4). The levels of the pain were recorded pre-operatively, at six hours, 12 hours, 24 hours, and 48 hours post-operatively based on the Visual Analog Scale/Numerical Rating Scale (VAS/NRS) score [50].

Serum markers

We hypothesized that the use of MINIMA® short stem would cause less bone damage comparing with the conventional Profemur® TL stem. Furthermore, we aimed to clarify whether the standard stem would have worse impact on muscle damage compared with the short one, taking into account that we performed the same approach for both cohorts. Therefore, we registered three common serum and inflammation markers, including C-reactive protein (CRP) [51], lactate dehydrogenase (LDH) [52,53] and creatine kinase (CK) [52,54]. Moreover, we attempted to record their interaction with other post-operative data. The serum markers were obtained and recorded pre-operatively as a baseline. Subsequently, they were registered 10 minutes after surgery, at first and second post-operative day.

Statistical analysis

First, Kolmogorov-Smirnov test was used to define the normality of the allocation of consecutive data. Normally allocated consecutive variables are demonstrated as mean ± typical departure. On the other hand, non-normally allocated variables are depicted as median and range of Q1 - Q3 quartiles. Explicit variables are indicated as total and pertinent (percentage) frequencies. Discrepancies in constantly and ordinarily allocated variables between the two femoral stems were collated using Student’s t-test. On the contrary, non-normally allocated variables were collated with the Mann-Whitney U test. Chi-square test and Fisher’s exact test were utilized to contrast discrepancies among categorical variables. Pearson or Spearman correlation coefficients tests were utilized to point out associations between continuous variables. Statistical significance was set up at two-sided p-value < 0.050 for all tests which were carried out. SPSS version 21.0 statistical software package for Windows (IBM - SPSS Inc., Armonk, NY, USA) was used in order to perform statistical analysis.

## Results

Patients’ features

The pre-operative demographic characteristics are demonstrated in Table 1.

**Table 1:** Pre-operative patients’ demographic characteristics in MINIMA and TL groups kg/m^2 ^= kilogram per square meter, g/dL = grams per deciliter

	Minima group	TL group	P-value
Number of patients	23	23	
Diagnosis			0.755
Osteoarthritis	8 (34.8)	9 (39.1)	
Dysplasia (all types)	9 (39.1)	10 (43.5)	
Other Osteonecrosis Protruzio	1 (4.3) 3 (13.0) 2 (8.7)	0 (0.0) 2 (8.7) 1 (4.3)	
Revision	0 (0.0)	1 (4.3)	
Age (years)	55±8	68±8	<0.001
Gender (female)	14 (60.9)	20 (87.0)	0.044
BMI (kg/m^2^)	32.15±6.28	32.86±5.01	0.676
ASA grade			0.002
Ι	9 (39.1)	1 (4.3)	
ΙΙ	10 (43.5)	21 (91.3)	
ΙΙΙ	4 (17.4)	1 (4.3)	
Charlson Comorbidity Index			0.201
1	21 (91.3)	23 (100.0)	
2	2 (8.7)	0 (0.0)	
3	0 (0.0)	0 (0.0)	
Pain	6.0±2.0	7.0±1.8	0.079
Comorbidities			0.155
Up to 3	22 (95.7)	19 (82.6)	
4 or 5	1 (4.3)	4 (17.4)	
Hemoglobin (g/dL)	14.0±1.7	14.0±1.3	0.843
Hematocrit (%)	42.2±4.4	42.5±3.5	0.848

The two cohorts had no statistically significant differences regarding diagnosis (p=0.755), with the higher prevalence of osteoarthritis or dysplasia, BMI (p=0.676), Charlson comorbidity index (p=0.201), the levels of the perceived pain (p=0.079) and the levels of hemoglobin (p=0.843).

Conversely, the patients in the minima group were statistically significantly younger (p=<0.001) compared with the conventional group. Furthermore, the conventional cohort included more women than the minima group (p=0.044). Finally, there was a recorded significant difference concerning the ASA score between the two cohorts (p=0.002). Specifically, in the minima group, 10 patients (43.5%) had ASA score II, while 21 patients (91.3%) had the same score in the TL group. 

Peri-operative data

**Figure 3:**
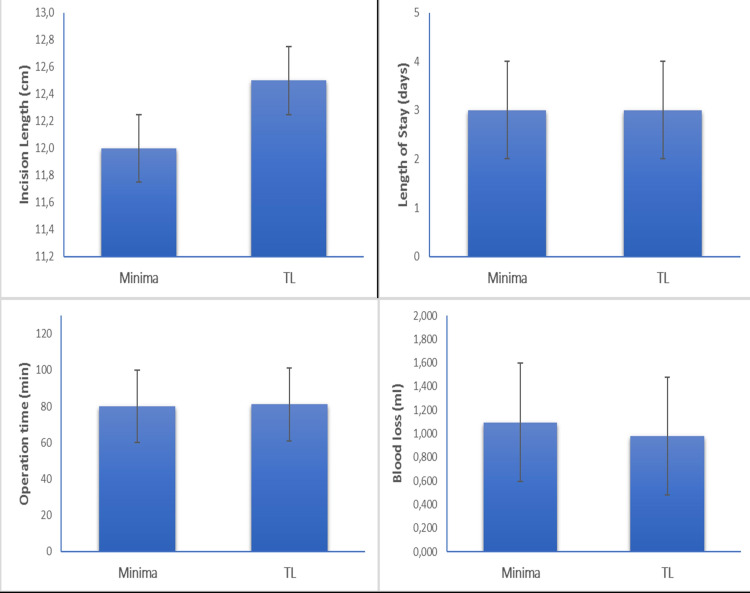
Comparison of peri-operative data between the two cohorts. All the discrepancies which are depicted have no statistical significance. cm = centimetre, min = minute, ml = millimetre

The collation of the peri-operative patients’ data among the two cohorts revealed similar rates of operative time (p=0.635), incision length (p=0.323) and length of stay (p=0.138). In the same way, no patient in our study required blood transfusion post-operatively. Unexpectedly, the patients with the MINIMA stem had a higher volume of peri-operative blood loss compared with the standard group but without notable discrepancies (p=0.067). Yet, this finding did not constitute a confounding factor for patients' hospitalization (Table 2, Figure 3).

**Table 2:** Peri-operative patients’ data NA = Non-available, min = minute, cm = centimeter, mL = milliliter

	Minima group	TL group	P-value
Operation time (min)	81±15	80±13	0.635
Incision length (cm)	12±1.5	12.5±1.4	0.323
Blood loss (ml)	1.096 (0.778 – 1.631)	0.980 (0.795 – 1.623)	0.067
Blood Volume change (mL)	1.096 (0.778 – 1.631)	0.980 (0.795 – 1.623)	0.067
Length of stay (days)	3 (2 – 4)	3 (2 – 4)	0.138
Anesthesia			0.265
Spinal	17 (73.9)	20 (87.0)	
General	6 (26.1)	3 (13.0)	
Transfusion rate	0 (0.0)	0 (0.0)	NA
Complications (yes)	1 (4.3)	2 (8.7)	0.155

**Figure 4:**
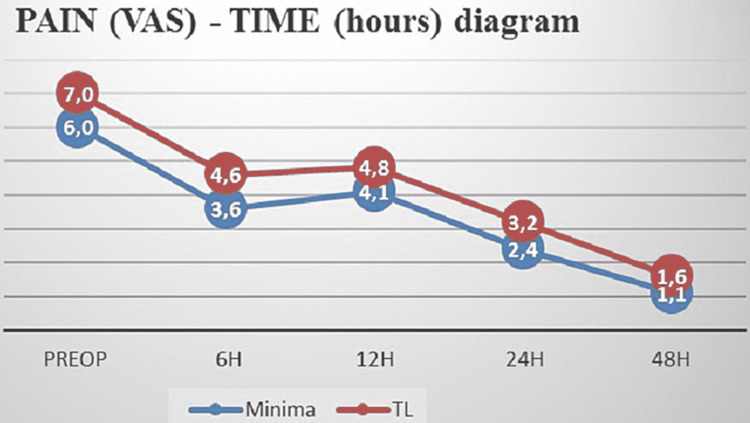
Pre-operative and post-operative pain levels according to Visual Analog Scale/Numerical Rating Scale in patients from both cohorts H = hours

On the other hand, patients in the minima group reported lower mean levels of the perceived pain all times post-operatively but without statistical significance (Table 3, Figure 4).

**Table 3:** Post-operative levels of perceived pain in patients undergoing total hip arthroplasty

	Time	Minima group	TL group	P-value
Pain	6 hours	3.6±2.7	4.6±2.1	0.184
	12 hours	4.1±2.8	4.8±1.9	0.335
	24 hours	2.4±1.8	3.2±1.7	0.108
	48 hours	1.1±1.3	1.6±1.0	0.165

Concerning complications, in the standard group, one patient was diagnosed with deep vein thrombosis (DVT) shortly after surgery and was administered the suitable treatment. In addition, one other patient was found with superficial wound infection two weeks after surgery and consequently underwent surgical debridement. On the contrary, in the minima cohort, one patient was diagnosed with superficial wound hematoma about 10 days post-operatively, which was dealt with surgical debridement. Thereafter, the clinical recovery was deemed satisfactory for all patients and no indications for revision surgery were registered. Finally, anterior thigh pain was not recorded in any patient of both groups.

Radiological outcomes

**Figure 5:**
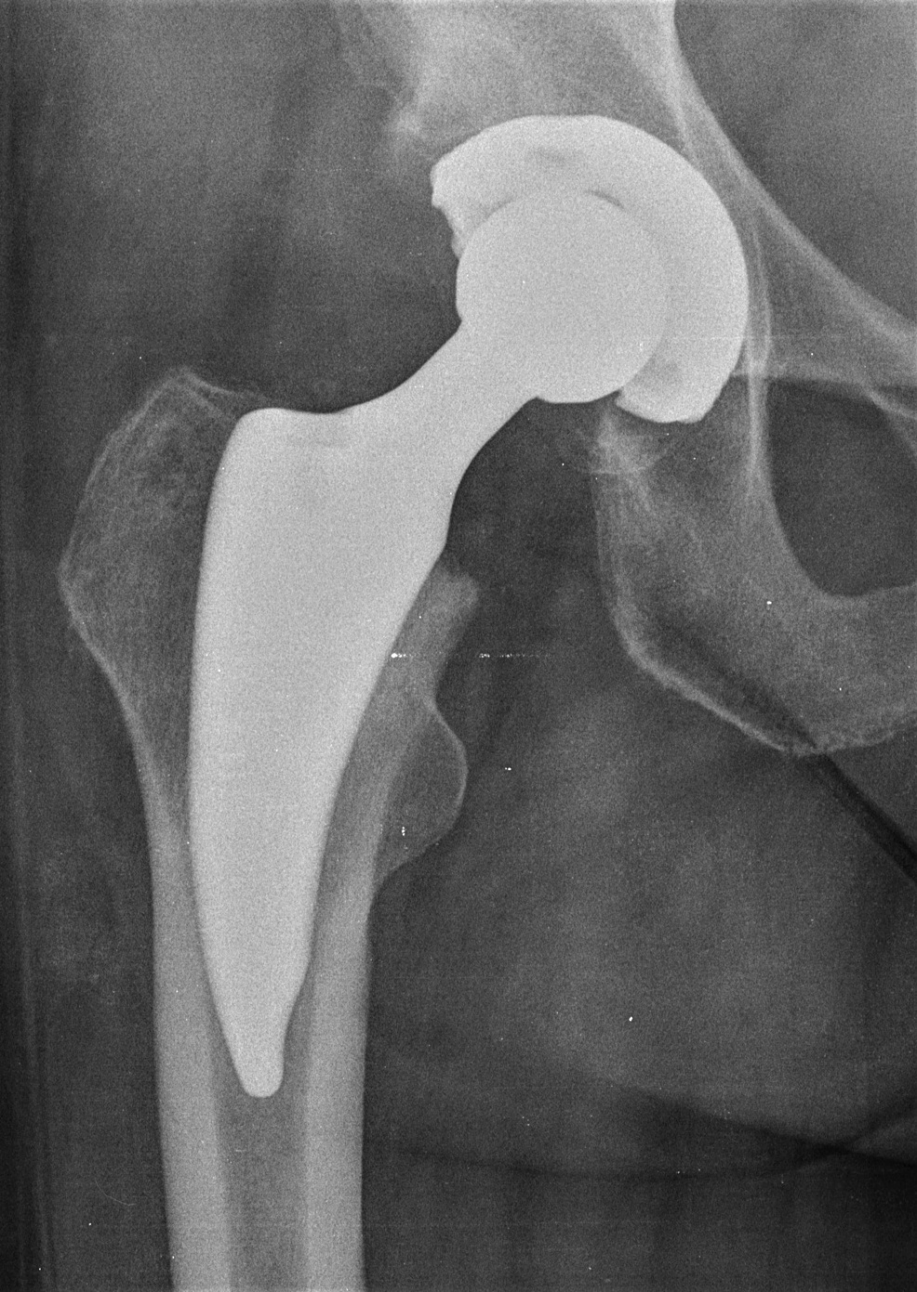
Post-operative x-ray of the MINIMA® short stem

**Figure 6:**
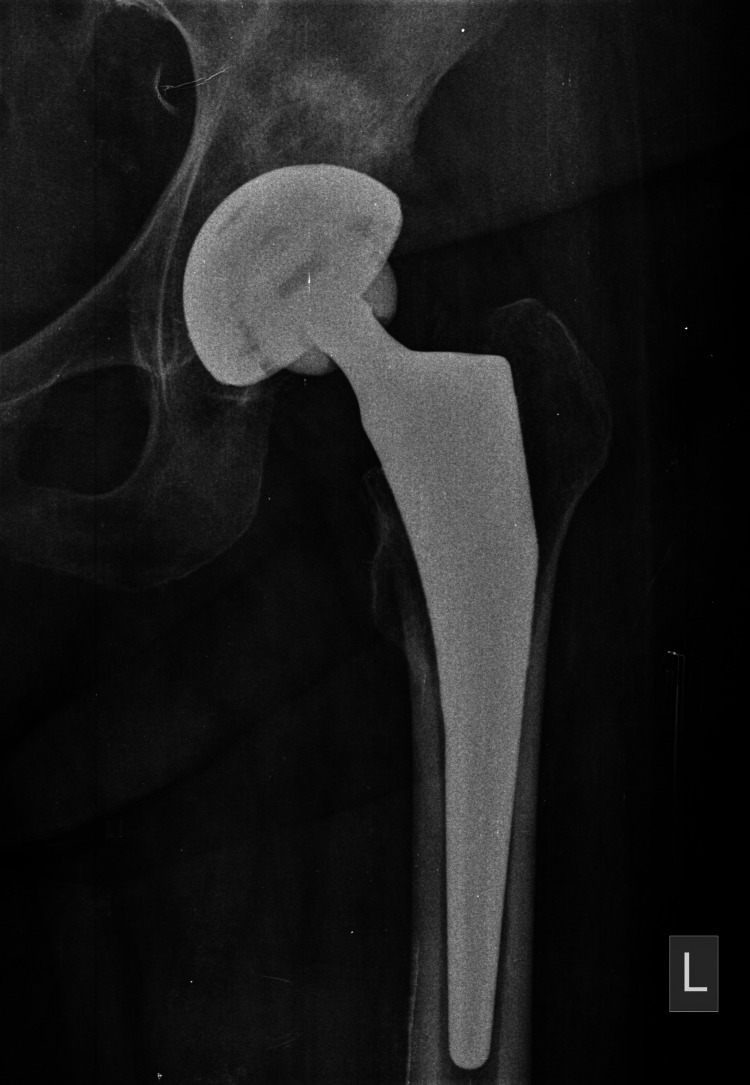
Post-operative x-ray of the Profemur® TL standard stem

The post-operative x-rays of the two stems are shown in Figures 5, 6.

Radiological evaluation revealed negligible discrepancies regarding the cup anteversion (p=0.535) and cup inclination (p=0.552) between the two cohorts and within acceptable limits. In addition, the cup component in all patients was regarded as stable and no radiolucent line came into view shortly (one year) post-operatively.

Similarly, the femoral stem was regarded as stable bone ingrowth for all patients from both cohorts one year after surgery. Moreover, no radiolucent lines or reactive lines, or cortical hypertrophy turned up in anyone of the 14 modified Gruen zones, while the femoral stress shielding and bone resorption fell into first degree for all patients from both groups.

The mean stem alignment fluctuation was measured within acceptable limits for both groups with similar results (p=0.351). For the minima cohort, the average leg length discrepancy was estimated (4.1±3.6 mm) and for the standard group was measured (3.1±2.5, p=0.282).

**Figure 7:**
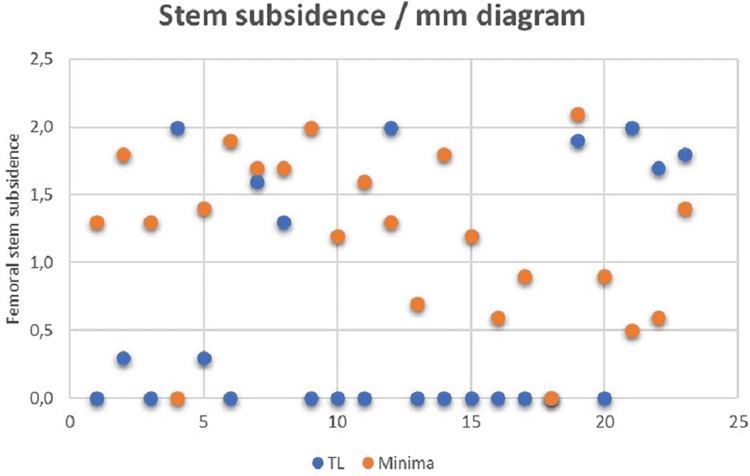
Femoral stem subsidence diagram in mm comparing the two different stems mm = millimetre

The mean femoral offset was recorded within accepted limits for all patients. Specifically, it was calculated higher concerning the minima group (44.6 mm) with a range of ±3.5 mm, while in the standard cohort, it was estimated 42.3 mm with a range of ±4.0 mm (p=0.047). In addition, significant lower femoral stem subsidence was recorded regarding the TL standard stem (0.6±0.9 mm) compared with the MINIMA short stem (1.2±0.6, p=0.026), but within acceptable limits for all patients (Figure 7).

Finally, no statistical difference was recorded between the two groups, regarding ectopic ossification (p=0.574) (Table 4).

**Table 4:** Radiologic evaluation of the position of the implants NA = Non-available; mm = millimeter; (x/y) % = minor and major axes' ratio

	MINIMA group	TL group	P-value
X-RAY - cup inclination / degrees	44.6±4.9	43.8±4.4	0.552
X-RAY - cup anteversion (x/y) %	23.5±8.8	25.0±7.9	0.535
CUP radiolucent lines (NO)	23 (100.0)	23 (100.0)	NA
CUP stability			NA
Stable	23 (100.0)	23 (100.0)	
Unstable	0 (0.0)	0 (0.0)	
Modified Gruen zones - radiolucent lines (NO)	23 (100.0)	23 (100.0)	NA
Modified Gruen zones - reactive lines (NO)	23 (100.0)	23 (100.0)	NA
Modified Gruen zones - cortical hypertrophy (NO)	23 (100.0)	23 (100.0)	NA
Femoral stem fixation			NA
Stable bone ingrowth	23 (100.0)	23 (100.0)	
Stable fibrous fixation	0 (0.0)	0 (0.0)	
Unstable fibrous fixation	0 (0.0)	0 (0.0)	
Ectopic ossification			0.574
Absent	18 (78.3)	18 (78.3)	
Grade I	4 (17.4)	5 (21.7)	
Grade II	1 (4.3)	0 (0.0)	
Grade III	0 (0.0)	0 (0.0)	
Grade IV	0 (0.0)	0 (0.0)	
Femoral stress shielding and bone resorption			NA
1st degree	23 (100.0)	23 (100.0)	
2nd degree	0 (0.0)	0 (0.0)	
3rd degree	0 (0.0)	0 (0.0)	
4th degree	0 (0.0)	0 (0.0)	
Femoral stem subsidence	1.2±0.6	0.6±0.9	0.026
Femoral offset	44.6±3.5	42.3±4.0	0.047
X-RAY -Leg length discrepancy /mm	4.1±3.6	3.1±2.5	0.282
X-RAY - stem degrees	1.6±0.9	1.3±0.8	0.351
Stem alignment (degrees)			0.291
Varus	11 (47.8)	6 (28.6)	
Valgus	10 (43.2)	15 (71.4)	

Functional and other scores

**Figure 8:**
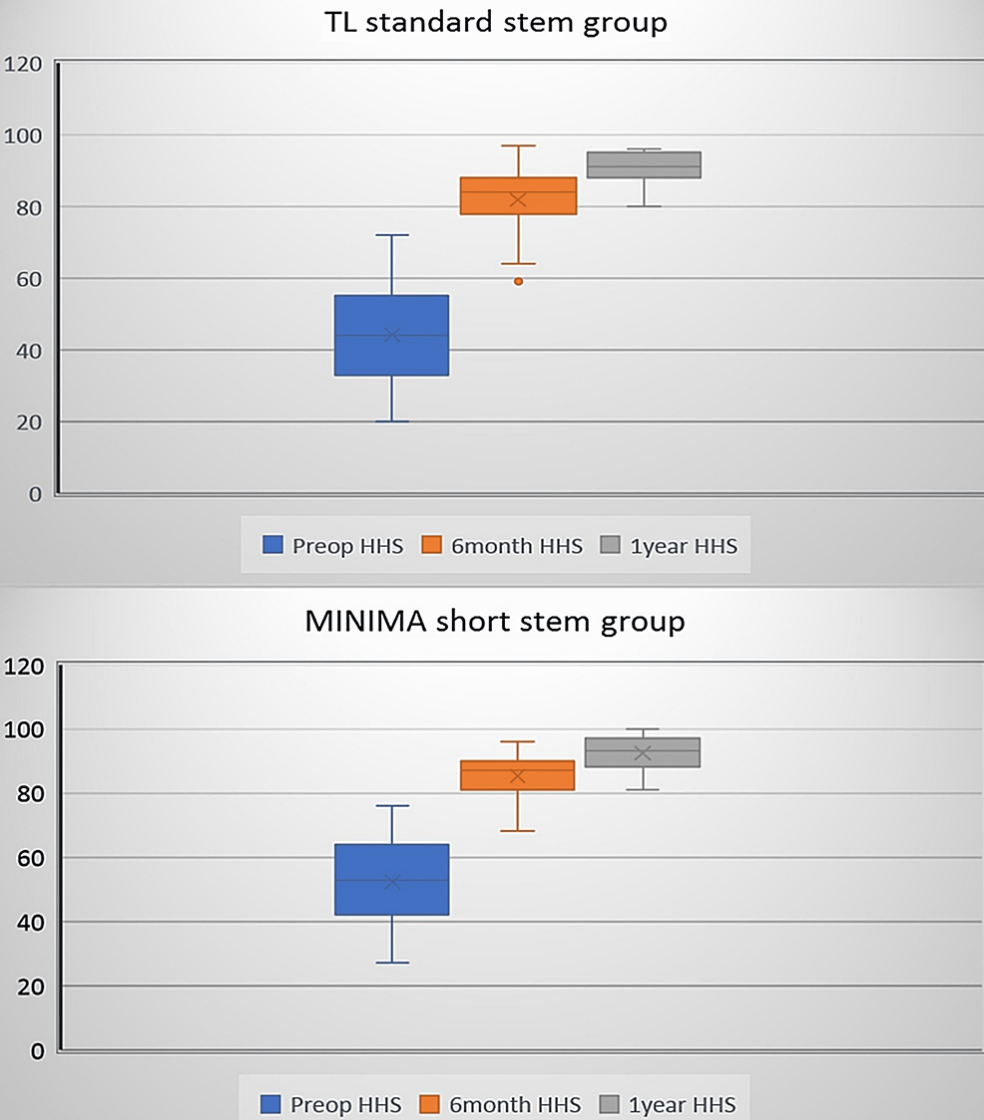
The functional Harris Hip Score (HHS), as it is recorded for both groups pre-operatively, six months and one year post-operatively

Pre-operatively, the average HHS was higher (52.3±14.2) in the minima group contrasted with the standard group (44.2±15.6), but without statistical significance (p=0.070) (Figure 8).

Similarly, the patients of the two cohorts were comparable concerning their clinical and mental status based on all the rest recorded scores.

At six-month follow-up clinical assessment, the patients in the minima cohort had definite better results in terms of Charnley hip score at the category of walking (p=0.008), EQ-5D score at the categories of self-care (p=0.015), usual activities (p=0.04), as well as anxiety/depression p=0.013. Regarding the levels of the perceived pain, although the mean and range values seem equal between the two cohorts, the statistical analysis revealed lower levels for the minima cohort.

At one year post-operatively, patients from both cohorts had substantially enhanced outcomes regarding all scores. However, statistically better results were recorded in the minima group concerning Charnley's hip score at the category of pain (p=0.048) and EQ 100 (p=0.041) (Table 5).

**Table 5:** Comparison functional patients’ data

	Minima group	TL group	P-value
Pre-operative			
Harris Hip Score	52.3±14.2	44.2±15.6	0.070
Charnley Score			
Pain	2 (1 – 4)	3 (1 – 4)	0.319
Activity	3 (2 – 5)	3 (1 – 5)	0.284
Walking	3 (1 – 5)	3 (1 – 4)	0.648
EQ-5D-5L			
Mobility	2 (2 – 3)	2 (2 – 3)	0.556
Self-care	2 (1 – 3)	2 (1 – 3)	0.724
Usual activities	2 (2 – 3)	2 (1 – 3)	0.660
Pain/discomfort	3 (2 – 3)	3 (2 – 3)	0.507
Anxiety/depression	2 (1 – 3)	2 (1 – 3)	0.819
EQ 100	69±12	66±15	0.391
DN4	2.5±2.1	3.2±2.0	0.257
PHQ9	3.9±3.3	4.9±3.7	0.340
6-month follow-up			
Harris Hip Score	85.3±7.9	81.8±9.3	0.182
Charnley’s Score			
Pain	5 (4 – 6)	5 (4 – 6)	0.025
Activity	4 (4 – 5)	4 (3 – 5)	0.154
Walking	5 (4 – 6)	5 (3 – 6)	0.008
EQ-5D-5L			
Mobility	1 (1 – 2)	1 (1 – 3)	0.686
Self-care	1 (1 – 2)	2 (1 – 3)	0.015
Usual activities	1 (1 – 3)	2 (1 – 3)	0.004
Pain/discomfort	2 (1 – 3)	1 (1 – 2)	0.670
Anxiety/depression	2 (1 – 3)	2 (1 – 3)	0.013
EQ 100	85±8	82±9	0.246
DN4	0.83±1.1	1.5±1.7	0.144
PHQ9	2.2±3.1	2.6±2.2	0.153
1-year follow up			
Harris Hip Score	92.4±5.7	90±4.9	0.186
Charnley Score			
Pain	6 (5 – 6)	5 (5 – 6)	0.048
Activity	5 (4 – 5)	5 (4 – 5)	0.976
Walking	6 (4 – 6)	5 (4 – 6)	0.181
EQ-5D-5L			
Mobility	1 (1 – 2)	1 (1 – 2)	0.813
Self-care	1 (1 – 2)	1 (1 – 2)	0.425
Usual activities	1 (1 – 2)	1 (1 – 2)	0.425
Pain/discomfort	1 (1 – 2)	1 (1 – 2)	0.680
Anxiety/depression	2 (1 – 3)	2 (1 – 2)	0.114
EQ 100	85±7	80±8	0.041
DN4	0.48±0.9	1.0±1.4	0.181
PHQ9	1.4±1.7	1.7±1.7	0.553

Serum and inflammatory markers

**Figure 9:**
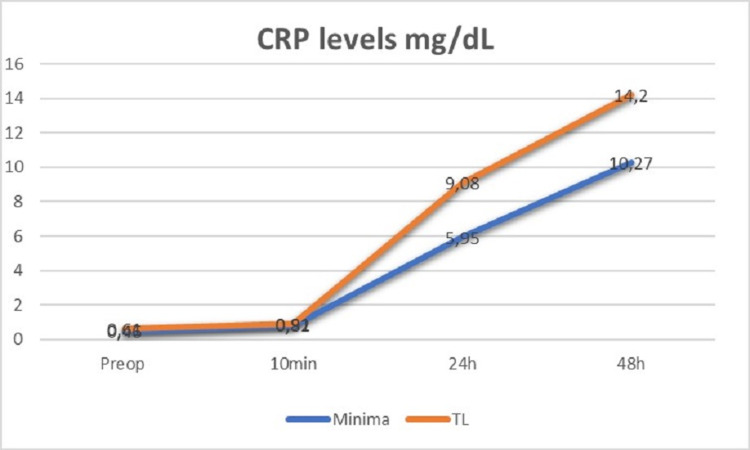
Perioperative CRP levels fluctuation for both cohorts. CRP levels were statistically significantly lower at 24 h and 48 h postoperatively in the MINIMA cohort mg/dL = milligram per decilitre, min = minute, h = hours

CRP levels were statistically lower in the minima group 24 h (p<0.001) and 48 h (p=0.004) post-operatively (Figure 9).

Similarly, LDH levels were lower in the minima group 24 h after surgery (p=0.007). On the other hand, no statistically considerable discrepancies were recorded concerning CPK levels at all times post-operatively between the two cohorts (Table 6).

**Table 6:** Peri-operative levels of serum markers min = minute, hrs = hours

	CRP			CPK			LDH		
	Minima group	TL group	P-value	Minima group	TL group	P-value	Minima group	TL group	P-value
Pre-operative	0.46±0.37	0.61±0.30	0.148	109.96±70.03	95.43±71.50	0.147	231.43±56.9	234.91±39.6	0.811
10-min post-operative	0.82±0.85	0.91±0.49	0.665	253.83±221.39	179.57±73.45	0.385	210.70±64.4	235.83±46.03	0.135
24-hrs post-operative	5.95±2.60	9.08±2.87	<0.001	823.43±454.73	827.17±537.97	0.852	211.13±69.03	262.91±54.8	0.007
48-hrs post-operative	10.27±4.96	14.20±3.83	0.004	933.09±717.39	792.83±408.61	0.767	234.74±80.45	227.30±80.31	0.755

## Discussion

A huge proliferation of THR amount has been performed worldwide, owing to life expectancy lengthening and patients’ demands. Furthermore, recent epidemiological studies underline the fact that the incidence of THR has been increased especially in young patients [18,55,56]. The biological behavior of the femoral component is determined based on several features which eventually consist of the so-called “stem identity.” These characteristics include the geometrical features (length, shape of the transverse cross-section), the material properties, and the surface material which settle on the biologic fixation [19]. The length of the femoral component consists of a crucial feature and plays a cardinal role regarding its location and orientation, stability, proximal bone loading, and the extent of bone stock damage [19,28]. Yet, a great deal of controversy surrounds the scientific answer about the ideal stem length [28].

As stated above, the use and study of short stems have been increased recently due to the benefits they may offer, especially in younger patients, including less stress shielding, proximal bone stock preservation, restriction of proximal-distal mismatch, and reduced thigh pain [18,19,20,24]. Concerning the survival rate of short stems, a recent review of clinical studies and national arthroplasty registries conducted by Hauer et al. (2018) and colleagues demonstrated a mean revision rate of about 4.8% 10 years post-operatively. These results are comparable with those of the Australian registry of short stems (6.6%), as well as with the arthroplasty registries regarding conventional femoral stems of Australia (5.1%), Un. Kingdom (5.2%) and N. Zealand (7.3%) [27].

The results of our study did not reveal noteworthy discrepancies between the two cohorts in terms of the operation time, incision length, blood loss, length of stay, complication rates, and the levels of post-operative perceived pain. Furthermore, the majority of the functional scores did not give strong prominence to one specific femoral stem, although MINIMA short stem was associated with relatively better post-operative functional outcomes. This outcome could be explained also by the smaller mean age of the patients of the minima cohort. Similar results were recorded in a more recent comparative meta-analysis regarding primary THA between 616 neck sparing short stems and 643 standard stems, conducted by Sivaloganathan et al. Specifically, they reported no statistically significant discrepancies in terms of functional outcomes, thigh pain, and dislocation or revision rates [57]. Another recent meta-analysis did not record significant discrepancies between short and standard femoral stems in terms of functional outcomes, except the anterior thigh pain which was associated with the conventional stems [26].

On the other hand, the literature highlighted the biomechanical theory that the more preserved neck the more challenging difficulties the surgeons have to encounter, including leg length discrepancy, the incorrect orientation of the components, and subsidence [58,59]. This fact might explain why type I short stems have been associated with high rates of complications and revision surgery [25,28]. A wide spectrum of different short stems is commercially available. Based on the existing literature, we have proposed a simplified classification of short stems in four types. Type 1 “column stem,” type 2 “partial column stem,” type 3 “trochanter-sparing stem,” and type 4 “trochanter-harming stem” including type 4a the “ultra-short stem” and type 4b the “short version of the standard stem” [18]. The MINIMA short stem which was applied to our patients falls into type 3 category and it is considered a trochanteric sparing stem.

Subsequent to THA, an alteration of the transmitted forces will come up [60]. This fact will bring on bone remodeling, known as “stress shielding.” Areas with raised exerted forces culminate in growth of bone density. Conversely, restriction of these forces come up with bone resorption and subsequently, aseptic loosening of the implant [60]. Consequently, initial load should be as much as sufficient, normal and balanced in all areas. This fact is of the essence regarding the stability of the implants and the final success of the operation.

Our results indicated the efficacy of both femoral stems. Specifically, they were considered one year post-operatively as stable ingrowth, with stress shielding and bone resorption at first category according to Engh et al. [45], without the presence of any radiolucent lines or reactive lines or cortical hypertrophy in anyone of the 14 modified Gruen zones. A recent meta-analysis by Liang et al. included 1,130 patients (1,387 hips) comparing short and standard stems in primary THA. Taking into account their outcomes, they recommended that SS may contribute to advanced bone remodeling and equivalent functional results contrasting with standard stems [61].

The extent of the stem migration shortly after surgery plays a major role concerning the long-term satisfactory outcomes [62-64]. Axial migration greater than 1.5 mm has been characterized as a risk factor for the implant breakdown [64]. The radiological assessment of the TL conventional stem revealed lower levels of stem subsidence compared with the MINIMA short stem one year after surgery, but within accepted limits for all patients, according to the literature. A radiological and clinical study conducted by Janke et al. studied 71 patients undergone THA with Metha® short stem (type II). They indicated caudal migration 0.73±0.99 mm one year post-operatively and 1.01±1.27 mm two years after surgery [60]. Another study conducted by Freitag et al. assessed the mid-term migration of a type III short femoral stem (Fitmore), applied in 73 patients. The subsidence was estimated approximately 0.7 mm one year post-operatively and 1.1 mm two years post-operatively [62]. Drosos et al. conducted a review regarding the evidence on primary stability based on the short stem type. Both clinical studies regarding stem migration, as well as in vitro studies concerning stem micromotion, are limited. Yet, they highlighted similar outcomes with conventional stems which have exceptional long-term results [34].

Our initiation to evaluate the bone impairment and muscle damage based on three common serum markers, revealed some advantages especially regarding CRP levels in the MINIMA group. This fact could be interpreted in light of the less bone damage that the MINIMA short stem causes comparing with the standard one. Furthermore, it may have a relative interaction to some extent with some categories of the functional scores post-operatively, but not with the other peri-operative data including post-operative pain. To the best of our knowledge, this is the first comparative study between a short and a conventional stem in primary THA which incorporated data of serum markers to evaluate bone damage and muscle damage in a single performed approach.

The outcomes of our comparative study should be interpreted in detail taking into consideration some limitations. Initially, our study was not a randomized clinical trial. Inevitably, the two cohorts were not comparable at the baseline especially regarding the mean age of the patients. Additionally, it included also a small number of patients of the two contrasted groups. Furthermore, the follow-up examination was recorded only at six months and one year post-operatively, including short-term radiological and functional results. Therefore, we cannot support the efficacy of this particular short stem compared with the standard one in the long term. In addition, the better functional outcomes regarding the MINIMA group in some scores post-operatively could be explained because of the mean younger age of the patients. Furthermore, the record of the bone damage and soft tissue impairment was based only in three common serum markers, taking into account that all the procedures were performed by the same surgeon (GD) with the same approach and similar operative time. The answers of which are the most suitable serum markers and which is the suitable time to evaluate them need to be clarified by the literature.

## Conclusions

In our comparative study, we aimed to investigate the potential short-term efficacy of the MINIMA short stem comparing with the conventional TL stem in primary THA. Both femoral stems were regarded as exceptionally efficient with great post-operative results. A postoperative outcome which captured our attention was the higher levels of some serum and inflammatory markers some time points after surgery concerning the MINIMA group. The outcomes which emerge from our study outlined the effectiveness of the new MINIMA short stem in primary THA, with comparable and, in some cases, relative better short-term results contrasting with the TL standard stem in the short-term. The long-term results remain to be scrutinized. Well-designed comparative studies with long-term results need to be conducted in order to further underline the potential efficacy of this particular short stem.
